# Conference Report: HYGROTHERMAL EFFECTS ON THE PERFORMANCE OF POLYMERS AND POLYMERIC COMPOSITES Gaithersburg, MD September 21–22, 1995

**DOI:** 10.6028/jres.101.077

**Published:** 1996

**Authors:** Martin Y. M. Chiang, Gregory B. McKenna

**Affiliations:** Polymers Division, Material Science and Engineering Laboratory, National Institute of Standards and Technology, Gaithersburg, MD 20899-0001

## 1. Introduction

This report contains the highlights of the presentations and discussions at the NIST/Industry/Academe Workshop: Hygrothermal Effects on the Performance of Polymers and Polymeric Composites that took place on 21–22, September 1995 at the Hilton Hotel, Gaithersburg, Maryland. The workshop was organized by the Polymers Division of the Materials Science and Engineering Laboratory (MSEL) at the National Institute of Standards and Technology. Sponsorship was provided by the Center for Theoretical and Computational Materials Science in MSEL. This workshop brought together 54 attendees; including 10 invited speakers, 15 representatives from the academic community, 18 companies and several Divisions within NIST, to define the state-of-the art in hygrothermal issues in polymers and polymeric composites.

The report is organized into four sections. Section 1 gives the objective of the workshop as well as outlines of the individual presentations and conclusions based on the discussions which took place at the time of the presentations. Section 1 also presents the accomplishments that resulted from the workshop. Section 2 contains the scope and conclusions of the individual presentations. In Sec. 3, we present a summary based on a survey of workshop participants and the general discussion that took place at the end of the workshop. Finally, we give the conclusions of the workshop in Sec. 4.

### 1.1 Workshop Objective

Polymers and polymeric composites are used successfully in myriad applications such as housewares, buildings, highway construction, electronics and aerospace, automotive, and transportation. However, the age-old problem of degradation over time after exposure to the environment will always be present. The implementation and usage of tailored engineering materials in structural and engineering designs require our understanding of material performance and ability to withstand environmental effects during long-term use. The goal of this particular workshop was to focus on the hygrothermal issues as related to environmental stability. The workshop was developed to bring together researchers from industry, NIST, and academe to assess the current state of theory and modeling of hygrothermal effects in polymers and their composites. In addition, the workshop addressed data needs and experimental capabilities and how they could be integrated with theory and modeling to enhance and promote our understanding of hygrothermal effects on the performance of polymers and polymeric composites.

### 1.2 Brief Outlines of Individual Presentations

The organization of the presentations in the workshop was divided into two major sessions. The first session focussed on *Academic Research* and the second session dealt with *Industry Perspective*. Each major session was divided into several subsessions, and each subsession was designed to have two presentations. *Academic Research* contained two subsessions: “Overview” and “Modeling.” *Industry Perspectives* accommodated three subsessions: “Challenges in Electronics Packaging,” “Characterization and Modeling in Microelectronics,” and “Challenges in Automotive Applications.”

Extended discussions followed at the conclusion of each subsession. These discussions provided the opportunity to address concerns and questions related to the relevant subsession. Furthermore, a 2 h general discussion was arranged at the end of the workshop, and a portion of this final discussion time was planned for those who had application concerns that did not fall into the above subsessions.

In the *Academic Research* session, Prof. Jack Weitsman of the University of Tennessee gave the first presentation in the subsession “Overview.” He reviewed the effects of fluids on the response of polymers and polymeric composites, based upon information gleaned from a survey of several hundred technical articles. What emerged from this review is that practical design considerations must derive from an adequate database that is specific to each material or material system. An assessment of the significance of fluid effects can be obtained by recording their weight-gain within the composites. Substantial departures from linear Fickian predictions portend serious degradation in mechanical properties, and should be followed by detailed study and microscopic examination.

Prof. Ken Ashbee of the University of Birmingham, UK, described osmosis and reverse osmosis phenomena in resin matrixes through the illustration of an experimental finding in 1748 by Abb’e Nollet. The flow of water through the membrane into the more concentrated solution can be prevented by applying a definite pressure to the solution; this pressure is called the osmotic pressure of the solution. When this pressure is reached there is equilibrium, that is, there is no further increase in the volume of water uptake. Experiments with cellulose acetate demonstrated that membranes became more impervious to solutes as the applied pressure was increased. Ashbee went on to show that this eighteenth century work is relevant to moisture-induced degradation of composites in the presence of mineral contaminants.

After the subsession “Overview,” Prof. Alexander Chudnovsky of the University of Illinois at Chicago commenced the subsession “Modeling.” He presented observations and modeling of stress corrosion cracking in engineering thermoplastics. An example of a reliability problem from the residential piping industry was shown. It was suggested that a thermodynamic model that accounts for coupling of various physico-chemical processes of material aging implemented in user-friendly software would lead to improved material design and selection based on reliability considerations.

Prof. Henryk Stolarski of the University of Minnesota ended the *Academic Research* session with a description of a finite element model, which combines diffusion and convection equations with large deformation elasto-viscoplasticity, utilizing concentration-dependent elastic and viscoelastic material properties to better represent the behavior of drying of thin films. He pointed out that the drying of polymer films often results in appreciable shrinkage. Even though drying usually occurs at elevated temperature, thermal effects on coatings contribute only a small amount of volume change compared to the shrinkage caused by the evaporation of solvent.

After the presentations of the *Academic Research* session, the workshop focus shifted to the session *Industry Perspective*. Dr. Luu Nguyen of National Semiconductor Corporation started the subsession “Challenges in Electronics Packaging” with a description of the challenges that hygrothermal issues present in microelectronics, especially in PEMS (plastic encapsulated microcircuits). The potential areas of research to achieve a better understanding and prediction of the effects of moisture were also highlighted. Dr. Mark Poliks of IBM Microelectronics presented new methods to locate the position and concentration of water within epoxy-glass circuit board composites during processing. The effects of moisture absorption and choice of coupling agent on the electrical and mechanical performance of the board were highlighted. New challenges for the circuit board industry were outlined: organic based chip carriers must pass more demanding testing originally intended for ceramic modules.

Next, in the subsession “Characterization and Modeling in Microelectronics” of Industry Perspective, Dr. Richard Cornelia of DuPont presented a description of the degradation of high performance composites by moisture. Although the application concerns were not directly associated with microelectronics, the fundamental hygrothermal issues in composites are strongly related. He showed that when polymers were exposed to cyclic wet/dry conditions, some polymers exhibited a substantial nonreversible decrease in the *T*_g_ (Glass Transition Temperature), which can be significantly lower than the dry *T*_g_. This is a process related to aging effects in polymers but which has not been investigated thoroughly. The result of the moisture cycling was to dramatically reduce the high-temperature serviceability of the composite. This new state is characterized by a lowered dry/wet *T*_g_, reduced mechanical properties and decreased limits of high temperature service. At the end of the first day of presentations, Dr. Richard Shook of AT&T Bell Laboratories showed that moisture level testing can be derated to various factory conditions through a moisture diffusion analysis, and a critical moisture accumulation at buried interfaces is an effective criterion for the derating approach.

The following day, the workshop started with the Industrial Perspective subsession “Challenges in Automotive Applications.” Dr. Elaine Yorkgitis of 3M Automotive gave the supplier’s view of the hygrothermal effect on automotive adhesive bonding with emphasis on epoxy-metal structural bonding. She described the causes of diminished durability of structural bonds and indicated that these are fairly well understood in a general sense when the bonding surface is a clean prepared metal. The overriding constraint for automotive applications of adhesive bonding, however, is that bonding often involves oily unprepared metal surfaces. For this situation, much still is not known. In the final presentation, Dr. Martin Trapp of Ford Motor Company gave the end user’s viewpoint on thermoplastic durability requirements due to hygrothermal effects for automotive applications (interiors and exteriors). The severity of automotive service environments for exterior and interior components including heat, moisture, and UV (ultraviolet) radiation were highlighted. He observed that, for example, in desert areas the temperature can exceed 114 °C in the interior of the vehicle—a temperature regime that is extreme for many engineering plastics. Also, he pointed out that hygrothermal effects are important factors that need consideration in the design of polymers and their composites for recycling.

### 1.3 Conclusions for the Discussions During Individual Presentations

It was clear from the presentations and discussions that, while there are specific problems associated with moisture in each industrial sector, there are also areas of common interest. A common problem that was recognized is that the impact of moisture on the mechanical response of polymeric materials is seldom fully characterized. Hence, there was a perceived need for better measurement procedures to obtain data that are relevant to the estimation of long term performance. Important common issues were the identification of changes in the *T*_g_ with moisture uptake (which affects service temperature because of changes in the viscoelastic response), changes in the fracture response due to plasticization or stress-cracking effects, and changes in the fatigue behavior of both polymers and composites.

In addition, the representatives from the electronics industry were concerned about the reliability of electronic systems that can be degraded during processing and use by moisture adsorption at the interface between the polymer and electronic components. Representatives from the composites industry demonstrated interest in interfacial effects and the automotive industry in adhesive fracture. Finally, there was general agreement that some of the problems in estimating the impact of moisture, either in general or at interfaces, arise from contamination of materials and interfaces by substances that adsorb or absorb moisture. Few studies of such effects have been made as related to these industries.

### 1.4 Major Accomplishments

This workshop identified moisture concerns in industrial materials and processes, determined scientific and technical needs, and provided input for developing models and better measurement techniques to address these issues. It also enhanced the interaction of NIST with the industrial and academic community. The workshop ended with an extended discussion and agreement to form working groups that address two of the identified areas of research: 1) Effects of moisture on the mechanical response of polymers with initial emphasis to be placed on viscoelasticity and fracture; 2) Influence of contaminants in the materials on the uptake of moisture and consequent deleterious effects at interfaces.

## 2. Summary of the Individual Presentations

### 2.1 Academic Research—Overview

#### Effect of Fluids on Polymeric Composites—A Review Jack Weitsman, University of Tennessee

Prof. Weitsman presented a review of the effects of fluids on the response of polymers and polymeric composites. His review contained a comprehensive listing of data regarding sorption processes that can be divided among several categories. Some categories are noted to be associated with reversible “benign” effects, while others are correlated with irreversible phenomena that lead to property degradation and failure.

**Fig. 1:**
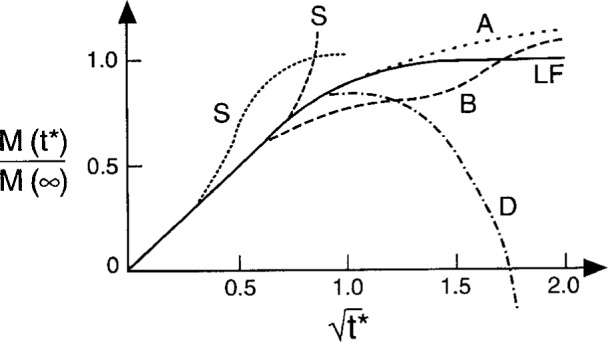
Schematic curves of non-Fickian weight-gain sorption data in polymers and polymeric composites. The solid line corresponds to linear Fickian diffusion. *M*(t^*^)/M(∞) is relative weight gain. t^*^ = *Dt/L*^2^ is the nondimensional time; *D* is the diffusion coefficient; *L* is the length and *t* is the time variable.

The kinetics of fluid sorption in polymers was discussed from a basic perspective: linear Fick’s Law, which predicts a reflective symmetry between mass-gain (typically, up to 8 % for technical polymers, 2 % for polymeric composites) in initially dry specimens and weight-loss data upon drying of saturated coupons (no hysteresis). He noted that, in many circumstances, weight gain data for the sorption and desorption of fluids in polymers do not concur with the prediction of Linear Fickian diffusion (Fig. 1, line LF). Schematic curves, shown in this figure, representing some categories of recorded anomalous sorption data (non-Fickian weight gain; lines A,B,C,D,S in Fig. 1) in polymers and polymeric composites were discussed. Some of these anomalies can be attributed to the inherent time-dependent response of polymers (viscoelastic diffusion). Also, moisture weight gain in polymeric composites exhibits departures from Linear Fickian behavior. Deviations from Linear Fickian diffusion behavior corresponds to the often encountered circumstance of a continuous gradual increase in weight-gain, never attaining equilibrium (line A in Fig. 1) or to the so-called “Two-Stage Diffusion” behavior (line B in Fig. 1). Circumstances where severe deviation from Fickian diffusion is observed usually correspond to a rapidly increasing moisture content within the composites. This increase is generally accompanied by noticeable degradation in properties, damage growth, material breakdown and/or mechanical failure. In addition, some of the severe deviations from Linear Fickian diffusion behavior correspond to weight-loss that can be associated with irreversible chemical or physical damage of polymer resins in conjunction with hydrolysis, or the separation of side groups from the polymeric chains, or the dissociation of material located at the vicinities of fiber/matrix interfaces.

Prof. Weitsman also emphasized that, although both the moisture content of polymers and temperature have qualitatively similar effects on the viscoelastic behavior of polymers, there is a vast difference in detail. This is because the diffusion processes and heat conduction occur on different time-scales, while viscoelastic response and moisture diffusion occur on similar time scales. Consequently, spatially nonuniform moisture results in spatially inhomogeneous creep. This inhomogeneity varies with time and results in a full coupling of two time-dependent processes.

Finally, what emerged from this review was the realization that the experience based on the above work can be used only as a guideline. In practice, design considerations must derive from a specific data base adequate to each case. Based on current understanding and modeling capabilities, it is very risky to extrapolate data in terms of time, temperature, stress, moisture, or other parameters.

#### Osmosis and Reverse Osmosis Ken Ashbee, University of Birmingham, UK

Prof. Ashbee discussed the importance of osmosis and reverse osmosis on the performance of composites in underwater applications. Osmosis is the flow through a semi-permeable membrane of solvent from dilute solution to concentrated solution, the two solutions being separated by the membrane. Such flow can be prevented by applying a pressure to the concentrated solution, this pressure is called the osmotic pressure associated with that solution. By applying still higher pressure, the flow can be reversed, that is, solvent can be made to flow from concentrated solution to dilute solution. This is known as reverse osmosis and is widely exploited for water desalination purposes.

**Fig. 2:**
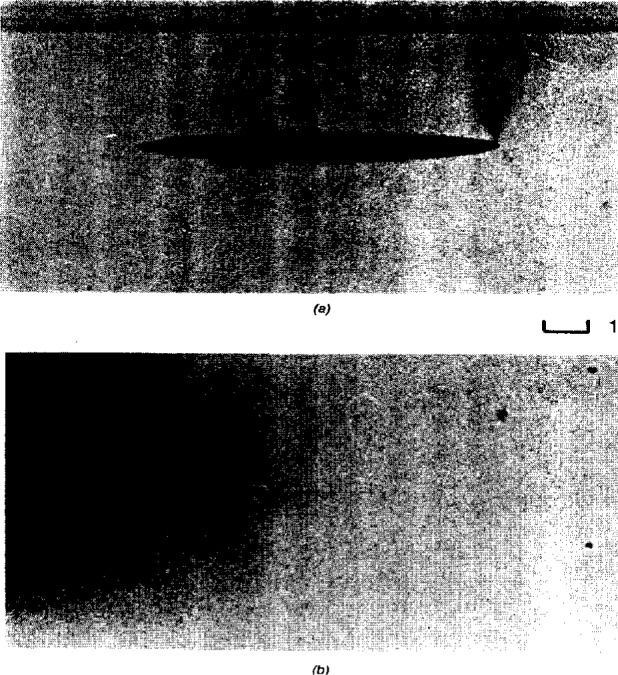
The elastic nature of osmotic pressure-filled penny-shaped cracks in epoxy in moist and after drying. Drying caused the crack faces to come back into contact with each other.

**Fig. 3:**
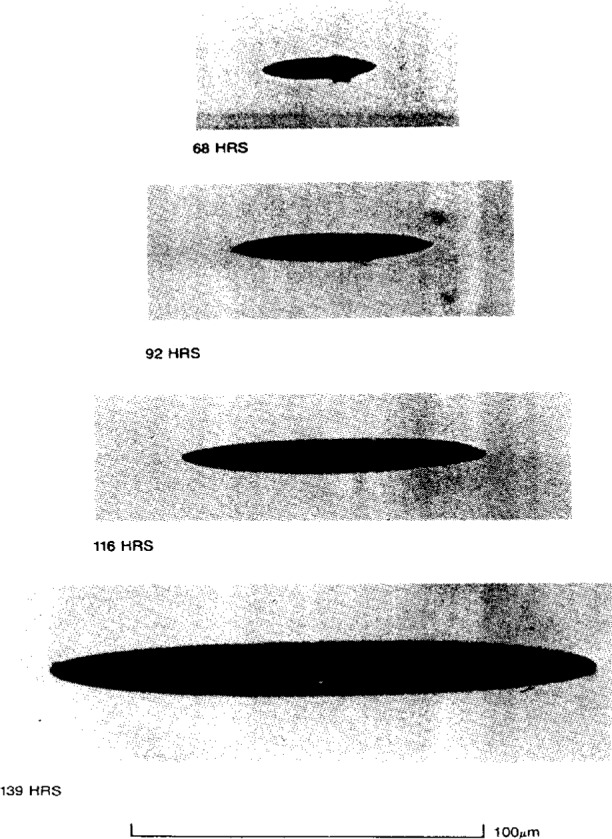
Edge-on views of a penny-shaped crack in an epoxy resin sample photographed at different times of the immersion in water at 94 °C.

Prof. Ashbee described that osmotic pressures are as high as 10 MPa to 30 MPa (several hundred bars) for saturated solutions of common inorganic impurities, and can give rise to the nucleation of pressure-filled cracks inside the polymer. Examples of such cracks in general purpose polyester and epoxy resins are shown in Figs. 2 and 3. The build-up of osmotic pressure depends on the difference in solute concentration either side of the semi-permeable barrier, and it is for this reason that the degradation in mechanical strength during water uptake by polymers is less severe in saline environments than it is in fresh water environments.

In as far as osmosis affects the modeling of water uptake, Prof. Ashbee drew attention to the fact that, when grown, the cracks shown in Figs. 2 and 3 are filled with aqueous solution. The solvent is free water. It is phase separated in the Willard Gibbs sense of phase separation, i.e., the individual volumes of solution are large enough for the Gibbs phase rule to be applied to them, and the distribution of this phase separated water is strongly inhomogeneous. In passing, Prof. Ashbee also mentioned other physical mechanisms for the existence of nonuniform distributions of diffused water. These include water uptake by polyester resins and by vinyl-ester resins accommodated by reverse esterification, i.e., by a nonuniform distribution of hydrogen-bonded hydroxyl radicals. Also, internal stresses, such as those created by polymer cure shrinkage adjacent to rigid filler particles and fibers, are relieved by, and therefore attract, diffused water. In the case of polyester and vinyl-ester resins, the same is true for internal stresses created by surface shrinkage due to post-curing following reverse esterification and to the leaching out of low molecular weight material. Yet another mechanism for the nonuniform accommodation of water uptake is water of hydration located at particles of filler. Turning to reverse osmosis, Prof Ashbee pointed out that, by applying to the solution a pressure in excess of the osmotic pressure, water can be made to flow across the semi-permeable barrier from concentrated to dilute solution. Water uptake by way of reverse osmosis poses a severe limitation to the application of polymers to deep sea submersibles. Reverse osmosis is expected at depths below about 250 m.

### 2.2 Academic Research—Modeling

#### Observation and Modeling of Stress Corrosion Cracking in Engineering Thermoplastics Alexander Chudnovsky, University of Illinois at Chicago

The key design parameters for durable applications (such as rigidity, yield strength, deformability, toughness, and glass-transition temperature) were addressed by Prof. Chudnovsky. He described that moisture uptake in materials, which contributes to material performance in durable applications, can cause the plasticization (softening) of polymers, the reduction of molecular weight due to chemical attack in polymers, the chemi-crystallization of polymers, the physical aging resulting in the build up of residual stresses in the polymers, the weakening of the interfaces between plastics and their substrates as well as morphological transformation.

**Fig. 4:**
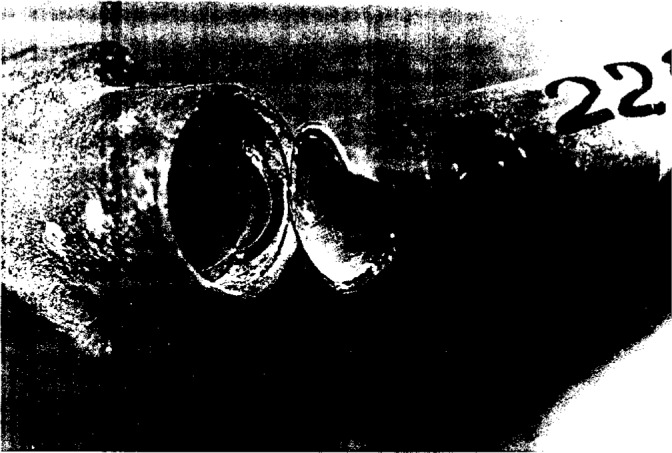
A general view of the fracture of PB pipe at the metal connector. The crack was initiated at the inner surface of the pipe exposed to a chlorinated water.

**Fig. 5:**
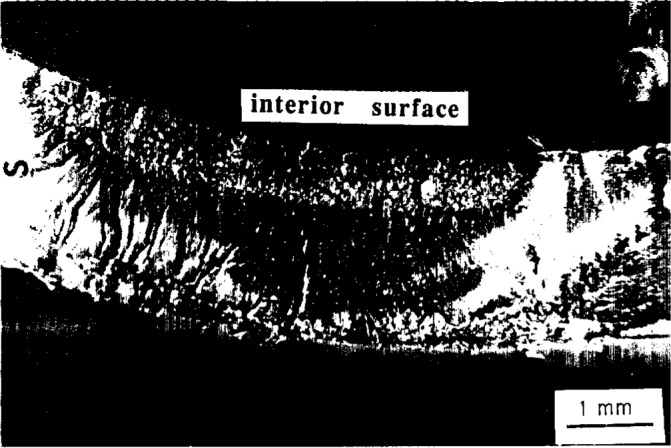
The pattern of striations shown in this figure is a typical one for a circumferential crack growing from inside out.

Several examples of field failure due to mechano-chemistry associated with moisture were presented. A particularly severe form of mechano-chemical degradation was described in case studies of premature failure of polybutylene (PB) piping system widely employed in residential plumbing application (Figs. 4 and 5). The premature failure of acetal fitting and polybutylene pipes is associated with mechano-chemical degradation highly accelerated by the presence of the chlorine in drinking water (Fig. 6). The critical issue in such cases is the prediction of the ability of the material to perform an intended function under specified service conditions. Therefore, due to the increasing usage of plastics in durable applications and the rising cost for potential product liability, there is a great need for methods to predict the expected lifetime of modern plastics in the anticipated environments.

He indicated that, in the past, the standard approach to accelerate a test for service life was to increase the stress (load) and/or to increase the temperature. Under such conditions, fracture occurred faster, but often by a different mechanism than observed in the field. As a result, researchers cannot extrapolate data from a short term test to the long term. However, a new criterion based on failure similar conditions offers the potential to replicate the field fracture and gives a more reliable estimate of lifetime.

He outlined the new method for predicting the reliability and lifetime of engineering thermoplastics. This method is based on the crack-layer kinetic model (a thermodynamic model) and computer simulation of various scenario of failure. Evaluation of the relevant material properties that account for the coupling of various physico-chemical processes is an essential part of the method. The comparisons between experimental observations and theoretical predictions, including the effect of mechano-chemistry on crack layer growth, were presented. In addition, he suggested that a fracture mechanism map instead of a single criterion is the first proper step toward material characterization for durable applications. Finally, he felt that NIST can provide its expertise in materials characterization and critical examination of the predictive fracture models.

#### Stress Effect in Drying Polymer Films Henryk K. Stolarski, University of Minnesota

In his presentation, Prof. Stolarski discussed the modeling of diffusion-induced stress effects in strongly deforming media. There are several industrial processes in which diffusion is accompanied by large deformations. The drying of protective, optical, or magnetic coatings (which results in large shrinkages) is one prominent example of such a process; thermal effects in casting and solidification may be another example (even though deformations are not necessarily very large in this case). Absorption of moisture (an effect opposite to drying) and resulting swelling clearly falls into the same class of problems and is important in many practical situations. In most of these processes, the material properties change appreciably with the solvent or moisture content; in thermal problems, they change with the temperature level. These quantities need to be known sufficiently well if meaningful estimates of stress and deformation fields are to be obtained.

In his presentation, Stolarski described the important phenomena in drying coatings (Fig. 7a). The diffusion and stress development during drying were modeled simultaneously to describe processes like those mentioned above. A large deformation multiplicative elasto/visco/plasticity was selected as a framework for the stress analysis. The elastic part of the model, based on nonlinear elasticity, is to accommodate nonlinearity typically present in polymeric materials. Changing material properties are simulated by evolving elastic parameters and by an expanding (or shrinking) yield surface, depending on local variations of solvent (or moisture, or temperature) level. This local level is obtained from the simultaneous solution of the diffusion problem that may include non-Fickian properties. Numerical solution of diffusion and stress development components of the problem are coupled because large deformations are allowed (Fig. 7b). Large deformations affect solvent concentration gradients and modify both the spatial and the temporal distributions of concentration.

Representative results illustrated the effects that the particular features of the model have on the final outcome of calculations. However, they are speculative in the sense that the required material properties, and particularly their variations with solvent concentration, are not known with any real accuracy. Nevertheless, it was thought that by presenting this development, and combining it with other contributions, practically meaningful models and calculations could be eventually made. To this end, improvements of the model based on a better understanding of the underlying material physics will be necessary. Furthermore, any associated experimental program will need to be designed to obtain material data based on the assumed model, i.e., it is important to obtain large-deformation responses for the material if these are what the model incorporates. The presentation made clear the need to coordinate experimental and modeling programs.

### 2.3 Industrial Perspective—Challenges in Electronics Packaging

#### Hygrothermal Effects in Plastic-Encapsulated Microcircuits Luu Nguyen, National Semiconductor Corp

**Fig. 8:**
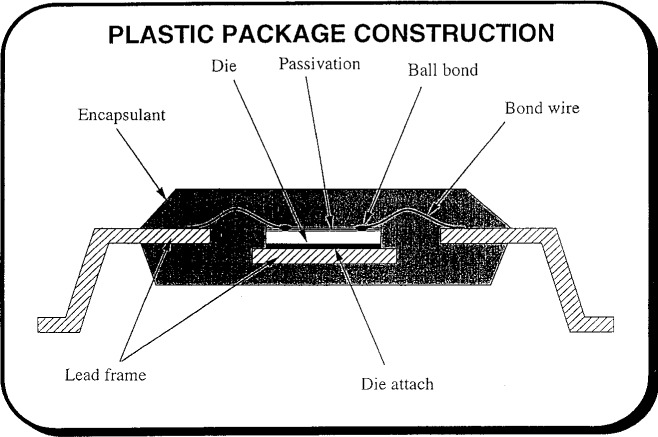
Typical construction of a plastic-encapsulated microcircuit (PEM).

In his presentation, Dr. Nguyen outlined the issues pertaining to hygrothermal effects in plastic-encapsulated microcircuits (PEMs, Fig. 8), and he highlighted some potential areas of research needed to achieve a better understanding and prediction of the effect of moisture on PEMs’ performance. A general background of PEMs and moisture induced package failure modes in PEMs was presented. Furthermore, the issues and challenges in electronic packaging due to hygrothermal effect (such as popcorning, moisture-induced device corrosion, long-term storage), rather than solved problems, were discussed. More than 90 % of the current worldwide usage of integrated circuits involves PEMs. With the current trend toward higher device functionality, circuit integration, and overall performance, even more stringent requirements are imposed on the PEMs. Constant enhancement in packaging materials technologies and processes have been made to address some, but not all, of these requirements.

One major problem that is still unsolved is the effect of moisture on the reliability of the device due to the hygroscopic nature of the typical epoxy molding compounds (EMCs). The amount of water absorbed by the package depends on a combination of parameters such as the form factor, the volumetric ratio of molding compound to leadframe metal and die, the formulation of the EMC, and the operating environment. This moisture, combined with the high temperature excursions that may occur during manufacture or service, can induce cracking in the packaging (popcorning). Although the existence of such cracks may not affect functionality immediately after assembly of the integrated circuits, the cracks introduce a path for ionic contaminants to infiltrate into the package and cause corrosion-induced failure during later service. Also, in a power IC device, such defects determine the potential for hot spots and increase the wearout rate of the device. With internal cracks, a high risk of failure exists if the operating environment requires repeated thermal cycling.

He also mentioned that with the increasing use of PEMs in military applications, another moisture-related issue is beginning to surface. Data on long term storage (e.g., 20 years) of PEMs under various field conditions are lacking. Similar to the observation of Cornelia (presented later), degradation mechanisms from repeated moisture absorption and desorption cycles may be important but are still uncharted.

#### Effects of Water in Epoxy-Glass Composites for Electronic Packaging Dr. Mark D. Poliks, IBM Microelectronics, Endicott, NY

Dr. Poliks’ presentation focused on the application of epoxy/glass composites in electronic packaging such as multi-layer first and second level electronic packages (chip carriers and circuit boards). Water in epoxy resins and epoxy/glass composites has been studied extensively at IBM. Absorbed water in resins and at composite interfaces can be a principal cause of conductive filament growth resulting in product failure. Conductive filaments form when defects at the resin/glass interface of the composite act as pathways for ion $\left({\mathrm{Cu}}^{2+},\;{\mathrm{Na}}^{+},\;K^{+},\;{\mathrm{Cl}}^{-},\;{\mathrm{SO}}_{4}^{2-},\;\text{etc}.\right)$ transport in the presence of moisture and bias. Surfaces of the glass fibers become plated with copper and result in internal electrical shorts.

**Fig. 9:**
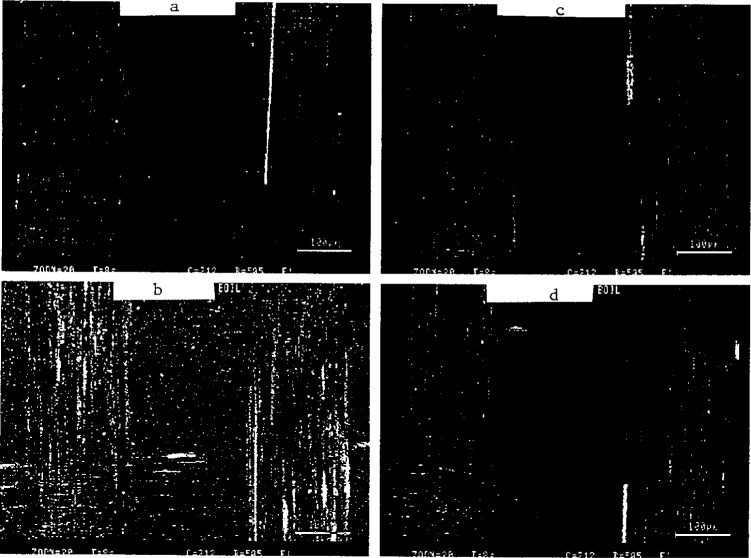
Laser Scanning Confocal Images of glass fabric reinforced FR4 (a,b) and Driclad (c,d) composites. Single ply laminates were exposed to boiling water for 4 hours (b,d). Refractive index oil (n d = 1.552) was used to improve the contrast between glass and resin. Regions of high contrast indicate separations between glass and resin on the order of 0.5 μm to 1.0 μm.

The water content and diffusion constants for brominated epoxy/glass system with material interfaces were described. Techniques used to study moisture and ions in composites include magnetic resonance imaging, dielectric spectroscopy, laser scanning confocal microscopy (Fig. 9), and laser desorption ion cyclotron resonance spectroscopy. In particular, a quartz crystal microbalance technique, in which mass is measured by monitoring changes in the frequency of an oscillating quartz crystal, was presented.

He presented moisture mass gain and diffusion coefficient data for two resin systems (FR4 and Driclad). It was found that FR4 absorbs about twice as much moisture in mass than Driclad even though they have similar diffusion coefficients. Moisture resistance of the material interface depends primarily on the chemical nature of the resin and coupling agent. The interface properties can affect the performance during assembly (e.g., delamination during soldering) and in-service reliability performance (internal shorts). By cross section, oxygen plasma etching and laser ablation, Poliks was able to examine single fiber glass/epoxy interfaces. He showed, by conclusive microscopic and chemical analyses, that moisture intrusion provides a mechanism for copper (Cu) deposition along the glass fiber. The mechanism and kinetics of conductive filament growth were also described.

### 2.4 Industrial Perspective—Characterization and Modeling in Microelectronics

#### Hygrothermal Performance of Polyamide Composites Richard Cornelia, DuPont

Absorbed moisture, present in all polymer matrix composites to varying degrees, plasticizes the resin, thus lowering the apparent *T*_g_ and, consequently, affecting the matrix-dominated mechanical properties. Upon drying, some systems are restored to their original dry *T*_g_, and this *T*_g_ is significantly elevated above the wet *T*_g_. However, some polyimides, when exposed to hot/wet conditions, have shown themselves to be morphologically metastable as evidenced by a steady decline in the dry *T*_g_. Physical aging involves changes in the physical structure and related properties of polymer glasses as a function of time. After typical processing, particularly with post-curing as an element, all polymer glasses, including polyimides, are kinetically arrested in an energy state above thermodynamic equilibrium. Exposures of polyimide resin composites (AFR-700B, Avimid® N, PMRZ15) to moisture caused a plasticization of the system, which decreased the *T*_g_ and correspondingly accelerated the relaxation toward thermodynamic equilibrium. Surprisingly, unlike prior experience, e.g., with epoxies, the lower *T*_g_ induced by the plasticization of the polyimides is not reversed upon drying and heating above the original *T*_g_. This permanent hygrothermal change of properties, unknown previously, critically reduces the upper service temperature relative to prior expectations based on non-cyclic hygrothermal exposure.

Additionally, Dr. Cornelia explained that the detrimental effects of ingress/egress of moisture on composites are generally underestimated by the industry. For example, he criticized the current view that in high speed civil transport (HSCT) applications composites can be modeled as dry structures. Although moisture levels entering flight may be low on a global basis, due to the diffusion process, surface plies can carry significant loads. Simulated flight cycles show that initial damage takes the form of transverse microcracks in these surface plies. This, in turn, promotes rapid/deeper moisture penetration with accumulating damage. Coupled with the *T*_g_ loss from hygrothermal effects, interior microvoids may subsequently appear and coalescence of damage can produce delamination. The degradation process is complex and depends on many factors such as environmental conditions, flight profiles, material type, thickness, *T*_g_, downtime, etc. Unknown synergistic effects will render modeling of this process particularly difficult.

#### Moisture Diffusion Analysis During High Temperature Solder Reflow of Plastic Surface Mount ICs Richard Shook, AT&T Bell Labs

This presentation addressed the issue of moisture ingress in plastic surface mount devices and its interdependency on solder reflow damage. Scanning acoustic microscopy was used to classify the moisture level in plastic electronic packaging. This moisture level testing can be “derated” to various factory conditions by using a moisture diffusion analysis. A moisture diffusion model, based on Fick’s equation, was developed and was used for the analysis of moisture diffusional kinetics during environmental exposures (moisture ingress) and during the solder reflow process (Fig. 10).

**Fig. 11:**
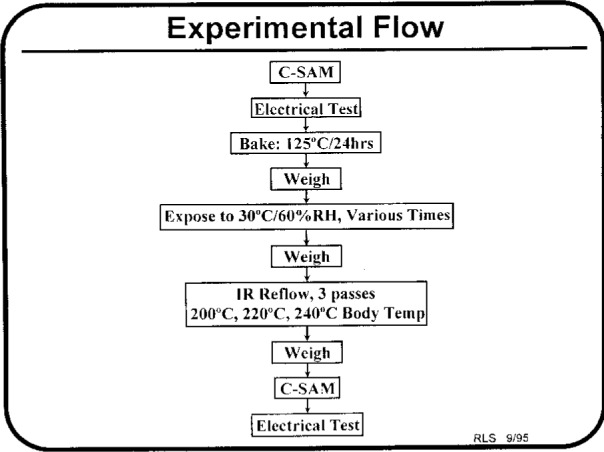
Experimental procedure verifying the effect of maximum reflow temperature on the moisture sensitive response of an 80-pin PQFP.

Information gained from the analysis of moisture accumulation at buried internal interfaces has led to a better understanding of the mechanism of moisture/reflow damage. A criterion based on the level of moisture accumulation at internal interfaces allows one to predict and extrapolate the expected reflow behavior to various combinations of temperature/humidity conditions. Verification of the predicted behavior, for different levels of the classification, was also performed through reflow experiments. Finally, the experimental procedure verifying the effect of the maximum reflow temperature was highlighted (Fig. 11 and Table 1).

### 2.5 Industrial Perspective—Challenges in Automotive Applications

#### Aspects of the Hygrothermal Durability of Epoxy-Metal Structural Bonds Relevant to Automotive Adhesive Bonding Elaine Yorkgitis, 3M

Dr. Yorkgitis gave an overview of the issues related to hygrothermal effects in automotive adhesive bonding. Typical uses of structural adhesives in automotive aluminum and steel bonding include frame bonding (primary structural bonding, Fig. 12a) and hem flange bonding (hoods, doors, and decklids, Fig. 12b). The general expectations of automotive users of structural adhesives were addressed within three broad categories—performance, process adaptability, and cost. These three categories also can serve as general guidelines and constraints for the development of automotive structural adhesives for aluminum and steel bonding.

The evaluation of adhesive performance in the noted application areas is based primarily on the adhesives’ ability to accommodate oil on the bonding surface, show adequate reactivity under the application conditions, provide and maintain sufficient strength, and exhibit the necessary failure mode, usually cohesive. Because factory conditions impose the overriding constraint that one is expected to bond to an oily unprepared surface, surface preparation was only briefly mentioned. The difference in attitude between the automotive and aerospace industries with regard to surface cleanliness and surface preparation was brought out using the example of aluminum substrates. The history, protocols, and recent advances regarding bonding to oily metal were addressed.

In consideration of adhesive-moisture interactions, the sources and control of moisture were discussed. The hygrothermal durability testing of automotive structural bonds can be divided into screening tests done by the supplier for development purposes and specification tests done by the supplier for product evaluation by the OEMs. In theoretical and experimental studies of hygrothermal durability of epoxy-metal bonds, the bulk properties of the adhesive have been studied in relation to bond surface and interface effects. Finally, the known and not (well) known causes of the many questions concerning diminished durability of structural bonds were discussed in Table 2. The development of structural adhesives which must accommodate oil but exclude moisture was presented as a challenge involving complex issues.

#### Thermoplastic Durability Requirements for Automotive Applications Martin Trapp, Ford Motor Company

As automotive companies continue to strive for the long-term durability of their vehicles, (currently at Ford new vehicles are required to meet or exceed 10 years or 150 000 miles) the need for hygrothermally stable thermoplastics will continue to rise. Understanding and predicting hygrothermal stability will be one of the keys in the design and development of such thermoplastics used in automotive interior and exterior (Table 3). By knowing “real world” automotive environments (heat, moisture, UV exposure, salt, dust, oxygen, ozone and microbiological attack) researchers are able to use accelerated techniques to predict long-term durability.

Dr. Trapp reviewed hygrothermal considerations (Table 4) and current accelerated methods (Table 5) used to predict extended durability in automotive applications. The general performance metrics are: no objectionable visual appearances (wrinkling, change in color, cracks …) on part surfaces, no dimensional instability that will interfere with normal functional operations, no loss of cold impact strength or coating adhesion. Also, thermal data from vehicles in Arizona, one of the more aggressive environments, were reviewed (Table 6). Automotive interior temperatures recorded in this high sunload region have been measured to be as high as 114 °C.

Finally, an analytical study of thermoplastic bumpers was presented to show how different real world environments could influence physical properties. Exterior bumpers recovered from vehicles after 3 years to 6 years in service show a reduction in impact properties (Table 7). In addition, it was pointed out that hygrothermal effects would influence the recycling characteristics of polymers and their composites.

## 3. Summary of Issues Survey for Discussions

To have an efficient discussion and to produce a more effective outcome, a questionnaire was distributed to workshop participants to help optimize the session discussions and formulate discussion issues.

This final discussion session described application concerns relative to hygrothermal effects on polymers and polymeric composites, identified the significant technical barriers concerning material characterizations, modeling and verifications, and determined serious obstacles that prevent further development in predictive capabilities. The workshop also identified key moisture related parameters and suggested the consideration of the synergistic effects of temperature, humidity and load in theory and modeling. The discussions resulted in an agreement to form working groups that address two of the identified areas of research: 1) Effects of moisture on the mechanical response of polymers with initial emphasis to be placed on experiments and modeling of resin viscoelasticity and interfacial fracture; 2) Influence of contaminants in the materials on the uptake of moisture and consequent deleterious effects at interfaces.

## 4. Conclusions

Industry representatives in fields such as R&D, material suppliers, end users, and engineering design identified their most pressing application concerns relative to hygrothermal effects on polymers and the most significant technical barriers that confront their applications. Researchers from academe, NIST, and private industry described the technical advancements or activities that have been done, the issues that needed to be addressed to overcome these barriers and the obstacles (current capabilities and limitations) that prevent further development.

From the presentations and discussions, it is clear that, due to the diversified nature of this workshop, there was no consensus on prioritizing the specific hygrothermal issues of polymers (or their composites) associated with each individual sector. However, the scientists and engineers participating in the workshop unanimously acknowledged the importance of fundamental measurements (fundamental understanding of material characterization) of hygrothermal effect on polymers that are relative to the modeling (predictive capabilities) and model validation. The majority of people at the workshop also admitted the significance of moisture effects on polymers over temperature, and identified important features of the synergistic effects of moisture and temperature and the synergistic effects of moisture and mechanical loads.

Based on the mission of NIST (which is to promote U.S. economic growth by working with industry to develop and apply measurements and standards) and its historical strength in fundamental studies measurement science, the workshop participants believed the government can play an important role in providing such fundamental needs and cohesion to the diverse industries, and ended with a recommendation to form future working groups that address the fundamental issues in 1) Hygrothermal effects on polymers and their composites, with initial emphasis to be placed on viscoelasticity and fracture; 2) Influence of contaminants in the materials on the uptake of moisture and consequent deleterious effects at interfaces.

## Figures and Tables

**Fig. 6a:**
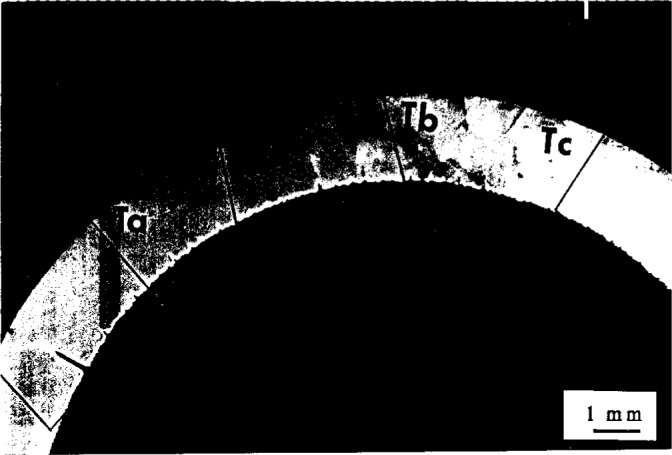
Shows a fragment of PB pipe used in residential plumbing. One can see a degraded inner surface (thin white layer) with longitudinal cracks almost regularly spaced.

**Fig. 6b:**
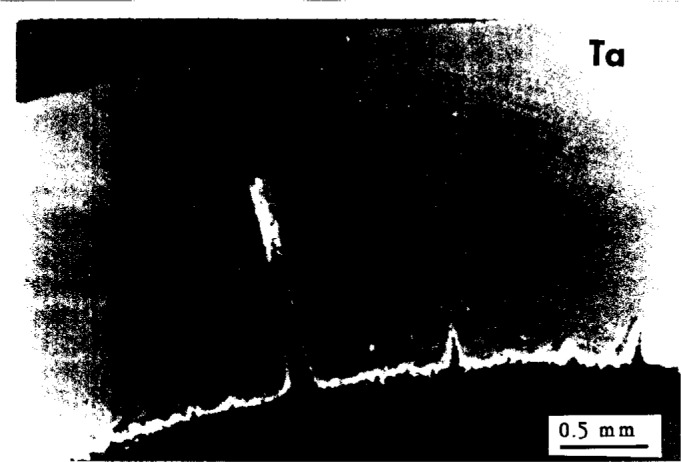
Displays a magnified view (Ta) of the largest crack with a zone of degraded polybutylene in front of it (crack layer). Although the pipe did not leak yet, the crack layer has penetrated about 2/3 of the wall thickness; thus the failure can be anticipated in a short time under regular service conditions.

**Fig. 7a:**
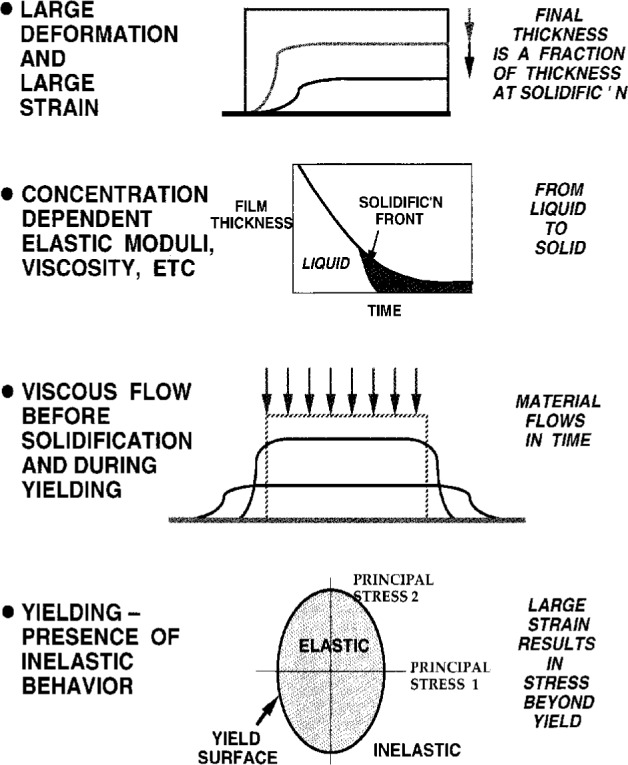
Important phenomena in drying coatings.

**Fig. 7b:**
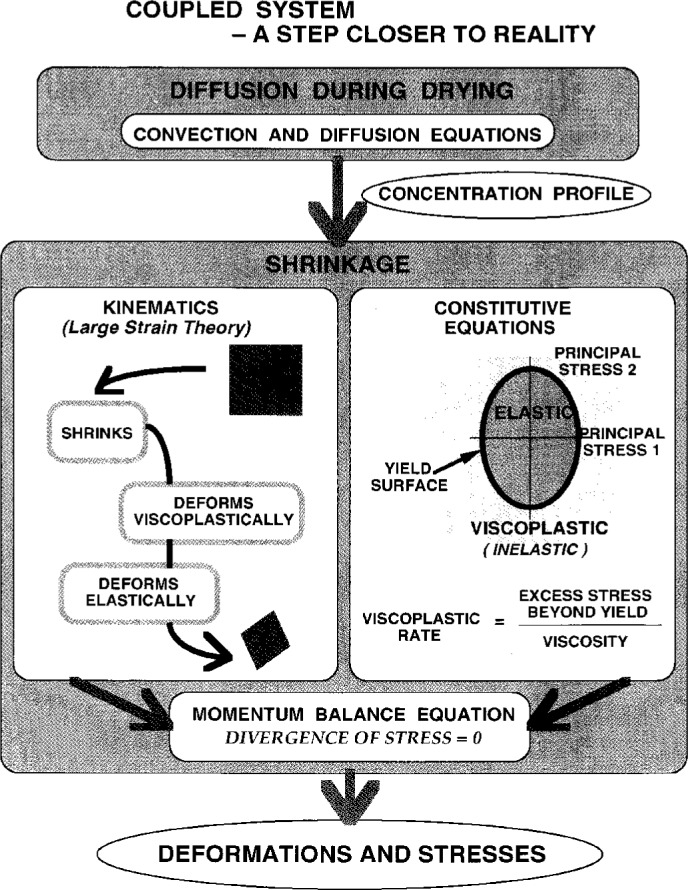
Basic features of the elasto/visco/plastic model used to describe stress development in drying materials.

**Fig. 10a:**
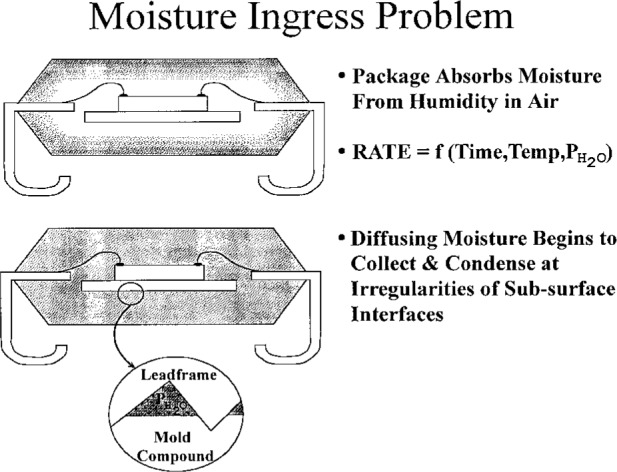
Schematic representation of moisture ingress of plastic surface mount integrated circuit packages.

**Fig. 10b:**
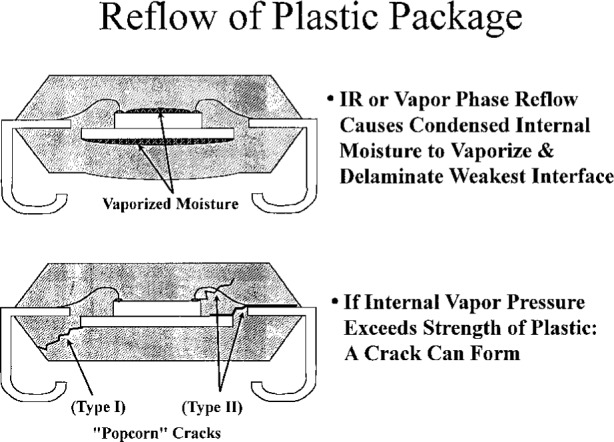
Schematic representation of reflow sensitive response of plastic surface mount integrated circuit packages.

**Fig. 12a:**
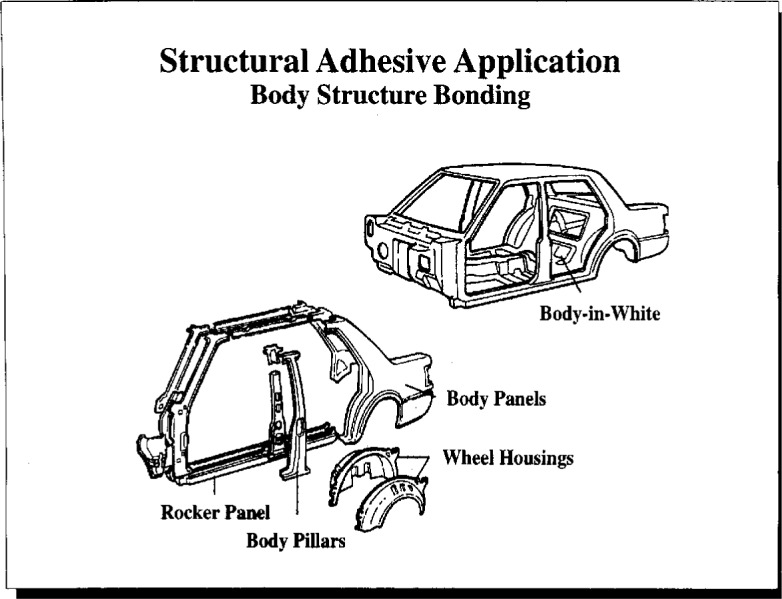
Primary strucural bonding in automotive aluminum and steel bonding.

**Fig. 12b:**
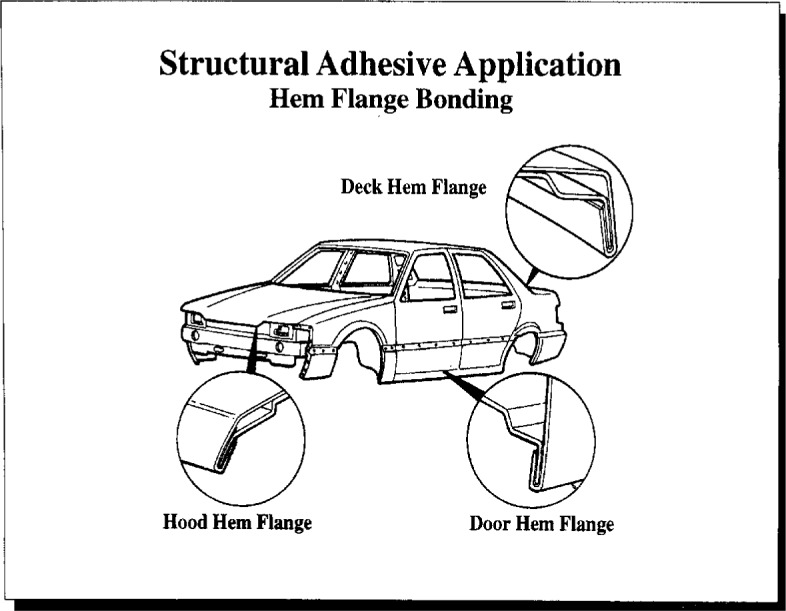
Hem flange bonding in automotive aluminum and steel bonding.

**Table 1:** Equivalent exposure times (hours) of an 80-pin PQFP @ 25 °C

	Percent relative humidity							
Max reflow temp.	30 %	35 %	40 %	45 %	50 %	60 %	70 %	80 %
200 °C	∞	∞	∞	∞	670	275	200	165
220 °C	∞	∞	∞	458	300	202	160	136
240 °C	∞	408	260	202	170	135	112	100

**Table 2:** Undelying causes of diminished durability of structural bonds

What is Known
Moisture and stress jointly and separately affect durability
Water reaches the interphase region via Diffusion through the bulk Passage along the interface and through the interphase region Direct wicking through any macroscopic flaws
Water will distribute itself through a bonded joint in a “redictable” fashion
What is Not (Well) Known
What is the interrelationship of diffusing lubricant and diffusion water in a structural joint? How does one account for the variety of lube types? Are there fundamental reasons to avoid water-based lubes and blank washes?
Is it truly desirable to remove all lubricant from the surface of the bonded steel or aluminum? Does the adhesive “replace” the tube? Is the adsorption of the lube any different than the adsorption of the adhesive? How is the surface best protected from water?
How does one reconcile the need to have a high-energy adhesive bond well to high-energy metals and the need to absorb oil from the metal surface so that the adhesive can reach that surface?

**Table 3:** Automotive material use and environment summary

• Thermoplastics are prevalent in the automotive interior and exterior, and their use will continue to increase (weight, cost complexity reduction (design for assembly/design for manufacturing), safety, …)	
• Interiors: ABS, PP, PC, PC/ABS, PA, POM, PPE, SMA, PBT, PET, TPOS, PVC, and TPE’s	
• Exteriors: SMC, ASA, PBT, PET, PA, PVC, ethylene ionomers, RIMPU, ASA/PC, ABS/PC, POM, TPE’s, TPO’s, and PC/PBT (PET)	
• Environmental factors:	Heat
	Moisture (dew, humidity, rain)
	UV exposure
	Salt, dust, ozone
	Microbiologic attack
• Paints: Enamel, lacquer, water base (epoxy, urethanes, and acrylics)	

**Table 4:** Hygrothermal considerations in automotive applications

**Moisture**
Water acts as a transport medium to bring oxygen in contact with the material leading to oxidation Parts in Florida see moisture ≈ 8 h/d, dew is saturated with oxygen and stays in contact with materials longer than rain Hydrolytic attack is accelerated with increasing temperature
**Temperature**
Surface temperatures can vary: Vehicle driven 90 km/is near ambient Vehicle parked in direct sunlight can be 30 °C above ambient At night vehicle surface can be 8 °C below ambient

**Table 5:** Current accelerated methods used to predict extended durability in automotive applications for high heat and sunload areas

Instrument panels and package trays Heat aging 500 h at 120 °C (Condition 24 h at 23 °C and 50 % relative humidity) Cold impact 24 h at 82 °C 5 h at – 30 °C (Condition 24 h at 23 °C and 50 % relative humidity) Rubber ball: mass = 4.54 kg, 127 mm in diameter, 60 durometer A, Height = 610 mm

**Table 6:** Ford—Arizona temperature study

• New River, Arizona	
• 1991 Thunderbird	
• 1993 Mark VIII	
• Temperature recorded from 7 am to 6 pm daily	
• Vehicles are on rotation turntables which track the sun	
• Data collected consists of:	Data, car angle, time, azimuth, altitude of sun, direct and diffuse solar intensity, wind speed and direction, outdoor relative humidity, interior vehicle temperature, exterior vehicle temperature, and shaded outdoor ambient temperature

**Table 7:** The reduction in impact properties of thermoplastic bumpers

• PC/PBT blend	
• Post consumer bumpers recovered after 3 years to 6 years in service	
• Physical properties evaluated:	notched izod impact strength melt flow rate molecular weight of PC
• Previous studies:	PC critical molecular weight = 33,800; after 5 years at 38 °C and 100 % relative humidity for a 50 % loss of tensile strength
	PC/PBT (50/50) blend 5 years to 6 years at 25 °C and 100 % relative humidity for a 50 % loss of impact strength

